# PSYCHIATRIC DISORDERS IN EARLY-ONSET VS. LATE-ONSET DEMENTIA: AN ANALYSIS OF THE ALL OF US RESEARCH PROGRAM

**DOI:** 10.1093/geroni/igae098.4256

**Published:** 2024-12-31

**Authors:** Xiang Qi, Ana-Maria Vranceanu, Bei Wu

**Affiliations:** New York University, New York, New York, United States; Massachusetts General Hospital, Boston, Massachusetts, United States; New York University, New York, New York, United States

## Abstract

Early-onset dementia (EOD), defined as being diagnosed with dementia before age 65, affects approximately 200,000 Americans. While EOD is characterized by an earlier and more severe onset, late-onset dementia (LOD) patients have longer exposure to potential risk factors for psychiatric disorders. This study investigates the relationship between EOD and psychiatric disorders, comparing it to LOD and individuals without dementia. We conducted a retrospective cohort study using electronic health records of 286,826 participants from the NIH All of Us Research Program. This diverse cohort included 59.7% women (mean age 52.3). Using multivariable logistic regression models, we found that compared to LOD, individuals with EOD showed increased risks of depression (OR 1.47, 95% CI 1.23-1.76), anxiety (OR 1.47, 95% CI 1.23-1.75), bipolar disorder (OR 2.63, 95% CI 2.04-3.38), schizophrenia (OR 2.76, 95% CI 1.96-3.88), and substance use and addiction (OR 1.86, 95% CI 1.96-3.88). The differences were more pronounced when comparing EOD to the no dementia group, with notably higher odds for depression (OR 4.82, 95% CI 4.37-5.33), anxiety (OR 3.79, 95% CI 3.30-4.36), bipolar disorder (OR 3.42, 95% CI 2.90-4.03), schizophrenia (OR 3.48, 95% CI 3.03-4.00), and substance use and addiction (OR 2.92, 95% CI 2.64-3.22). These findings persisted after controlling for sociodemographic characteristics, lifestyle factors, functional ability, and chronic conditions. Our results highlight the need for comprehensive mental health screening and targeted interventions for individuals with EOD. This study emphasizes the importance of early detection and specialized care for this vulnerable population, potentially informing healthcare policies and improving patient outcomes.

